# Apportionment and districting by Sum of Ranking Differences

**DOI:** 10.1371/journal.pone.0229209

**Published:** 2020-03-23

**Authors:** Balázs R. Sziklai, Károly Héberger

**Affiliations:** 1 Institute of Economics, Centre for Economic and Regional Studies, Budapest, Hungary; 2 Department of Operations Research and Actuarial Sciences, Corvinus University of Budapest, Budapest, Hungary; 3 Institute of Materials and Environmental Chemistry, Research Centre for Natural Sciences, Budapest, Hungary; Mathematical Institute, HUNGARY

## Abstract

Sum of Ranking Differences is an innovative statistical method that ranks competing solutions based on a reference point. The latter might arise naturally, or can be aggregated from the data. We provide two case studies to feature both possibilities. Apportionment and districting are two critical issues that emerge in relation to democratic elections. Theoreticians invented clever heuristics to measure malapportionment and the compactness of the shape of the constituencies, yet, there is no unique best method in either cases. Using data from Norway and the US we rank the standard methods both for the apportionment and for the districting problem. In case of apportionment, we find that all the classical methods perform reasonably well, with subtle but significant differences. By a small margin the Leximin method emerges as a winner, but—somewhat unexpectedly—the non-regular Imperiali method ties for first place. In districting, the Lee-Sallee index and a novel parametric method the so-called Moment Invariant performs the best, although the latter is sensitive to the function’s chosen parameter.

## Introduction

Comparing apples and oranges is never easy. But what if we are forced to do so? Fair division methods are hard to compare as each one was designed with a different goal in mind. One way to deal with the problem is axiomatic analysis. Finding out which method satisfies which fairness properties and make a choice based on this analysis. Policy makers, however, might need to evaluate the efficiency of their measures or need to justify their decisions by providing some numerical evidence. Thus, another stream of literature focuses on quantifying different aspects of the methods and comparing them numerically—Sum of Ranking Differences (SRD) follows this path.

The aim of this paper is twofold. Firstly, to promote SRD, a novel statistical method which is rapidly gaining popularity in various fields of applied science, such as analytical chemistry [1, 2], pharmacology [3], decision making [4], and finance (see case study No. 2 in [5]) and which can be also potentially interesting to the Political Science and Social Choice community. Secondly, to use this method to analyse two notoriously divisive issues related to proportional representation. These two issues represent two typical problem sets in social choice literature: fair division and fair assessment problems.

SRD allows us to select the most adequate solution among outcomes with different features based on a reference point. This situation is very common in multiobjective optimization, where the decision maker has to choose between many possible Pareto-optimal outcomes [4]. The problem analogous to fair division, where solutions satisfy different sets of fairness criteria. Since these criteria are usually conflicting, meaning there is no universally best solution, the decision maker has to choose one among them.

There are a number of problem instances where this kind of analysis can be valuable. Possible applications include, among others, the comparison of resource allocation schemes (*e.g*. cooperative game theoretical solutions, cake cutting rules) and the ranking of different measures (*e.g*. voting power indices, centrality measures). Thus, SRD can be applied to a wide variety of problems. Here we demonstrate its usefulness by ranking the solutions of two frequently studied problems in Political Science: apportionment and districting. The first can be characterized as a fair division problem while the second belongs to fair assessment. Ricca *et al*. [6] provides a very nice introduction to both topics.

The idea of proportional representation is prevalent in parliamentary democracies. Elections are considered fair if each voter has approximately the same amount of influence on the outcome. In most democratic countries, some members of the House are elected directly in single member constituencies. These constituencies are created by dividing up larger administrative regions, *e.g*. counties or states. To ensure equal representation, the seats of the House have to be distributed among these administrative units in proportion to their population. To be more precise, some countries consider total population, while others the number of voters. In some cases these base numbers are further adjusted (*e.g*. with the area of the county) to compensate for other factors. Most notably, rural areas are often treated better, in order to avoid a situation where the Members of the Parliament represent only a geographically small part of the country. In any case, the sizes of the constituencies have to be more or less the same. What makes this task difficult is that allocating a fixed number of seats among counties of different sizes often leads to divisibility issues. As fractional seats cannot be allotted, we have to decide which county gets more and which one gets less seats than its fair share.

Legislative bodies, both in the US and in Europe advocate that proportionality should be the key factor in apportionment. In the US, the 14th amendment already established proportionality as a fundamental principle [7]. In Europe the Venice Commission, the advisory body of the Council of Europe in the field of constitutional law also attested that equality of voting power should be achieved by creating constituencies of equal size (Venice Commission [8], Section 2.2, §13-15).

Even-sized constituencies are a necessary but not sufficient condition for proportional representation. Elections are often manipulated by gerrymandering—the redesign of constituency boundaries with the intention to favour one of the parties. Ansolabehere and Palmer [9] find that 20% of the congressional districts of the US remarkably lack compactness, while The American Prospect reports that “Close to a hundred congressional seats and thousands of state legislative seats have been strategically drawn to be noncompetitive at the expense of all other interests” [10]. As a result some constituencies obtain an unnatural, grotesque shape. Constituency boundaries may be affected by the geography of the region, by administrative or historic boundary lines, or because of the concentration of a specific national minority, but often the sole reason of districting is to manipulate the outcome of the election. Hence, there is a fine line between districting for valid reasons and gerrymandering.

To combat this weakness, US states impose a number of standards for redistricting. In addition to contiguity requirements, many states have a compactness clause in their election law, some even prescribe basic compactness tests. For a complete list, see [11]. Still, most statutes are so vague, that it makes it very difficult to challenge the design of constituencies at court based on compactness issues alone. For example, Idaho [12] requires “To the maximum extent possible, the plan should avoid drawing districts that are oddly shaped.”. Although many algorithms have been proposed to mathematically define and measure compactness, it is not yet clear which approach is the most relevant one.

The structure of the paper is the following. In Section 2, we describe the methodology, we introduce the SRD method and give a detailed example. In Section 3, we analyse the apportionment and districting problem through case studies. Finally, we conclude by pointing out interesting future research directions.

## SRD

Sum of ranking differences is a simple but effective statistical tool to rank and numerically assess different solutions based on a reference [13, 14].

The input of an SRD analysis is an *n* × *m* matrix, where the first *m* − 1 columns represent the different models (measurement techniques, Pareto-optimal outcomes), while the rows represent the measured variables (properties). In the following we will refer to the columns as *solutions*, and the rows as *objects*. The last column of the matrix has a special role. It contains the benchmark values, called *references*, which form the basis of comparison. From the input matrix we compose a *ranking matrix* by replacing each value in a column—in order of magnitude—by its rank. Then SRD values are obtained by computing the absolute differences between the column ranks and the reference ranking and summing them up.

### Reference values

SRD requires a reference value for each object. In some cases, justified reference values are available (prescriptions, earlier measurements). In the absence of a known gold standard, these reference values have to be extracted from the data. This step is called the *data fusion* [15]. Depending on the type of data, this can be done by a number of ways. Here we list the most common methods.

- Average (arithmetic mean). Not only the random errors but the systematic ones (biases) of different methods, and/or different measurement techniques follow normal distribution. If average is used in the data fusion act, the errors cancel each other out supported by the maximum likelihood principle and empirical evidences [16].
- Minimum/maximum. Error rates, residuals, misclassification rates, *etc*. often can be grasped with the minimum values. Row maximum is a suitable gold standard for the best classification rates, correlation coefficients *etc*. Row maxima and minima should be chosen whenever objects are maximized or minimized under optimal conditions. Such a selection of a benchmark is equivalent to defining the hypothetically “best” method with the smallest error, best classification, *etc*.
- Median. A “self-evident” substitute for the mean for asymmetric distributions, in the presence of outliers.

### SRD step by step

SRD is not solely a distance metric, but a composite procedure including data fusion and validation steps. Here we describe how it works in details. In addition, SRD is summarized on an animation procedure as a supplementary file in Bajusz *et al*. [17]. An SRD toolbox in MS Excel macro format is available at: http://aki.ttk.mta.hu/srd.

1. Data fusion: The definition of a reference (benchmark) depends on the features of the data set. The background philosophy is similar to proficiency testing (interlaboratory comparisons), where laboratories and techniques are compared using Z-scores with the assumption of normality [16]. Reference is either a known gold standard, or computed row-wise as the function of the first *m* − 1 column values.
2. Converting the data matrix: We create a ranking matrix by replacing each value in the column by its rank. That is, for each column (including the reference) take the smallest value in the column and replace it with ‘1’, take the second smallest value and replace it with ‘2’, and so on. Finally, the last remaining value, which was the largest of the original column values, is replaced by ‘*n*’. Ties in column vectors are resolved by giving the same rank to cells with the same value: the arithmetic mean of the ranks. This tiebreaking mechanism is called *fractional ranking* and is the standard in all statistical tests that deal with rankings.
3. Computing the SRD values: We calculate the (absolute) ranking differences between the reference and solution vector coordinates and sum them up. The SRD values are, in fact, city block (Manhattan) distances, and they rank the solutions. The smaller the SRD value the closer the solution is to the benchmark, *i.e*. the better. The mutual proximity of SRD values indicates the specific grouping of variables.
4. Validation: To remain comparable within various data sets (and different number of rows) the normalized SRD values (scaled between 0 and 100) are calculated. The permutation test (also called randomization test, denoted by CRRN = comparison of ranks with random numbers) shows whether the rankings are comparable with a ranking taken at random or they are different from it significantly. The second validation option is called cross-validation, and assigns uncertainties to the SRD values. Leave-one-out cross-validation is applied if the number of rows is less than 14. Leave-many-out cross-validation is applied for larger number of rows in the input matrix.

### Validation

SRD values follow a discrete distribution that depends on the number of rows. If *n* exceeds 13 the distribution can be approximated with the normal distribution well. The difference is already negligible for *n* > 10, but for values *n* ≤ 13 the SRD distribution is provided in the SRD toolbox. By convention we accept those solutions that are below 0.05, that is, below the 5% significance threshold. Between 5-95% solutions are not distinguishable from random ranking, while above 95% the solutions seem to rank the objects in a reverse order (with 5% significance).

The second validation step is cross-validation, where we repeatedly compute the SRD values, while one seventh of the objects is left out. This can be done in blocks or by selecting random rows. If the number of objects is small only one row is left out in each step. The median of the normalized SRD values are computed for each solution. The medians are then compared with Wilcoxon matched pair signed rank test (henceforward Wilcoxon test) to obtain a grouping of the solutions. The Wilcoxon test is a non-parametric statistical hypothesis test that can be used to compare two related samples. It is a common alternative to the paired Student’s t-test (also known as “t-test for dependent samples”) when the sample size is small and the population cannot be assumed to be normally distributed.

Cross-validation is similar to a Monte Carlo simulation where we randomly generate data and test the different methods. Generating random data might be difficult as the underlying distribution is often unknown, thus new, smaller datasets are produced by sampling the rows. Note, that we did not make any assumption on the independence of the objects. Indeed, SRD works fine even when there is some dependency between the objects. Cross-validation, however, might contain some noise if the solutions are not consistent. A solution is inconsistent if upon receiving a sub-set of the objects as input, assigns different values for those objects, than what the solution prescribed for the same sub-set for the original problem. This noise can be eliminated by computing the solutions for the smaller problems during each step of the cross-validation.

### Example

Now we demonstrate how SRD values are computed. Table 1 compares a couple of mobile phones based on the technological benchmark values of six features (Battery, Performance, Storage, *etc*.). In order to compare the features, the benchmark values are normalized. Note that this example is illustrative—in the case studies, the objects we analyse are of the same kind. Reference indicates the desired parameters. Phone A has a little more battery life and better camera than Phone B, but inferior in other aspects. First, we compute the ranks for both of the phones and the reference values. The smallest number in the column of Phone A is the RAM, so it will be the first in the ranking. The second smallest number in the column is the CPU performance, which therefore is ranked second, and so on. Notice that in case of Phone A, display and storage tie for the 3rd and 4th place, thus they each get an average rank of 3.5. Similarly, in case of Phone B, storage and RAM ties for 4th and 5th place, so they get an average rank of 4.5.

**Table 1:** Calculation of the SRD values.

Features	Phone A	Rank	Diff.	Phone B	Rank	Diff.	Reference	Rank
Battery	0.814	5	0	0.793	3	2	0.750	5
Performance	0.661	2	1	0.700	1	0	0.594	1
Storage	0.681	3.5	0.5	0.844	4.5	0.5	0.719	4
Camera	1.000	6	0	0.975	6	0	1.000	6
RAM	0.587	1	2	0.844	4.5	1.5	0.703	3
Display	0.681	3.5	1.5	0.709	2	0	0.625	2
SRD values			5			4		

After we computed the column ranks, we compare them with the reference ranking. SRD values are obtained by first taking the absolute difference of a column ranking with the reference ranking objectwise, then summing up the differences. In the example, Phone B is somewhat closer to the expectation, than Phone A.

Table 2 demonstrates the steps of the Wilcoxon test. Each row represent an SRD computation for a sub-set of the objects. As *n* is small, leave-one-out cross-validation is applied. That is, the Gr1 row was obtained by leaving out the first row, Gr2 by leaving out the second, and so on. The *Diffs*. column shows the differences of SRD scores, while the next column their absolute value. The latter is then used to create a ranking, tiebreaking is again resolved by fractional ranking. Finally, we reapply the signs, that is, ranks that originated from a negative difference are multiplied by (−1).

**Table 2:** Cross-validation—The computation of the Wilcoxon test.

Samples	*SRD*_*A*_	*SRD*_*B*_	Diff.	Abs.	Unsigned ranks	Signed ranks.
Gr1	5	1	4	4	6	6
Gr2	3	4	-1	1	2	-2
Gr3	4	2	2	2	4.5	4.5
Gr4	5	4	1	1	2	2
Gr5	1	2	-1	1	2	-2
Gr6	2	4	-2	2	4.5	-4.5

The last column is used to calculate the test statistics, *W* which is the minimum of two values: the sum of positive ranks (*W*_+_) and the sum of negative ranks (*W*_−_). *W* follows a specific distribution with an expected value of zero and which for large *n* converges to normal distribution. In the example, *W* = *min*{6 + 4.5 + 2, 2 + 2 + 4.5} = 8.5 under which we reject the null-hypothesis and conclude that Phone B is closer to the reference.

**Fig 1:**
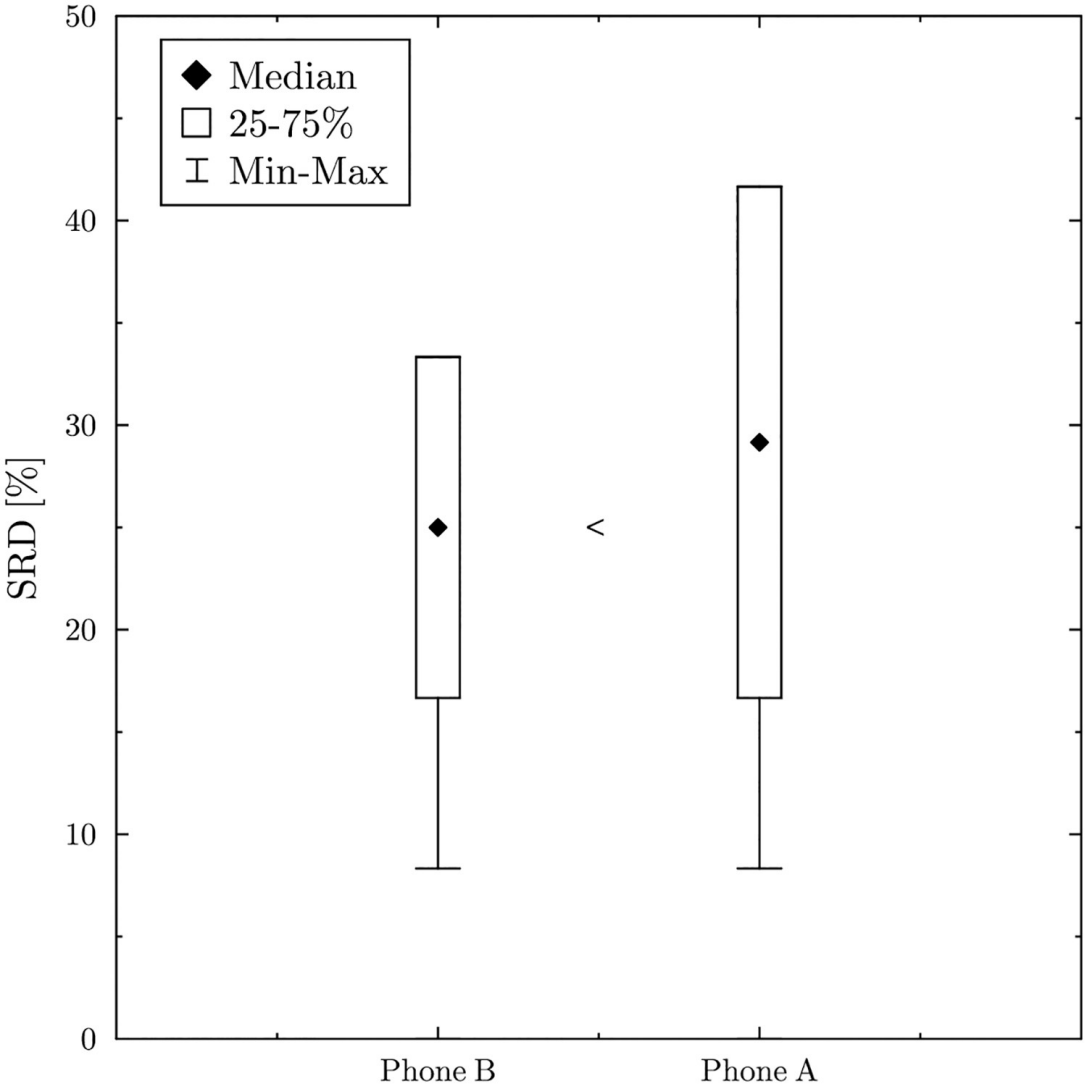
Cross validation—‘<’ indicates that the solutions significantly differ (at the 5% level) according to the Wilcoxon test.

The results are visualized in Fig 1. The boxplots represent the the first two column of Table 2, that is, how the SRD scores ranged in the cross-validation. For comparability reasons, SRD scores are normalized with the maximum possible difference, which is 12 for 5 objects. The whiskers indicate the minimum and maximum values, in case of Phone A these are $0.8\bar{33}=1/12$ and $0.41\bar{66}=5/12$. The boxes indicate the range between the first and third quartile. Note that, since in this example we had only 6 data entries for each solution, the 2nd and the 5th largest values were chosen as the first and the third quartile. The Wilcoxon test tells us whether the difference between the boxplots is significant.

## Case studies

In this section we demonstrate how SRD can be helpful in fair division and fair assessment situations. To be conform with the terminology of the apportionment literature, in the mathematical description we will use ‘state’ instead of ‘county’.

### The apportionment problem

In the apportionment problem we have a finite number of seats, which have to be distributed among states with different populations. The problem is analogous to the distribution of seats between parties, which received different number of votes during the elections. Brill *et al*. [18] also showed that many apportionment methods can be formulated as multiwinner approval rules. The US was the first modern country that adopted sophisticated apportionment techniques. Balinski and Young [7] give a comprehensive historical overview of the theoretical and political debate that surrounded the introduction and evolution of apportionment methods in the US. Here we restrict ourselves to discussing the main challenges and the proposed solutions that emerged in the past two centuries. First, we introduce some notation.

#### Mathematical framework

Let *m* denote the number of states in the country. An *apportionment problem* is a pair (***p***, *H*) that consists of a vector ***p*** = (*p*_1_, *p*_2_, …, *p*_*m*_) of state populations, $p_{i}\in {\text{I}\;\text{N}}_{+}$ and a positive integer $H\in {\text{I}\;\text{N}}_{+}$ denoting the number of seats in the House. An *apportionment method* determines the non-negative integers *a*_1_, *a*_2_, …, *a*_*m*_ with $\sum_{i=1}^{m}a_{i}=H$, specifying how many seats each of the states 1, 2, …, *m* obtains. Formally, it is a function *M* that assigns an allotment for each apportionment problem (***p***, *H*). Note that apportionment methods usually do not include any tiebreaking mechanism. A general assumption in the literature is that all the *p*_*i*_ values are different, which is virtually always true for real instances. Furthermore, let $P=\sum_{i=1}^{m}p_{i}$ denote the total population of the country, and let $A=\frac{P}{H}$ be the average size of a constituency. We refer to the fraction $\frac{p_{i}}{P}H=\frac{p_{i}}{A}$ as the *respective share* of state *i*.

#### Properties of apportionment methods

Apportionment rules can be classified into three categories: largest remainder methods, divisor methods and optimization methods. Each of the three approaches possess some unique trait that the others do not.

One of the most basic properties of apportionment is the so-called *Hare-quota*: if exact proportional allocation of the seats is not possible due to divisibility issues, it is reasonable to find an allotment nearest to the respective shares of the states. Formally, each state should be allotted at least as many seats as the lower integer part of its respective share (lower quota). Conversely, no state should obtain more seats than the upper integer part of its respective share (upper quota). When an apportionment method satisfies both upper- and lower-quota, we say it has the Hare-quota property.

Largest remainder methods were designed to exhibit this property. The best known such method is the Hamilton-method (sometimes also called Vinton-method), which first assigns each state its lower quota, then the remaining seats are distributed one-by-one to the states with the largest fractional parts of their respective shares. The Droop method is calculated in a similar way, but the states’ respective share is obtained by dividing the state populations with $\left(1+\frac{P}{1+H}\right)$. This results in different lower quota, and different fractional parts.

Hamilton-method, as all largest remainder methods, is vulnerable to monotonicity issues, which was the main reason why it was abandoned by US legislators. The most famous monotonicity paradox is the Alabama-paradox. Statisticians observed, that increasing the House size sometimes result in less seats for some states. Another paradoxical phenomenon is when a dynamically growing state is losing seats against a state with smaller population growth [19]. Hamilton-method is neither House- nor population-monotone. In addition, it also suffers from the New State and Elimination paradoxes (see Balinski and Young [20] and Jones *et al*. [21] for further details).

Divisor methods are immune to monotonicity paradoxes. A divisor method is characterized by a monotone increasing function f:IN→IR, the so-called *divisor criterion*. The $\frac{p_{i}}{f(s)}$ value is the *rank-index* or *claim* of state *i* when it has *s* seats. Seats are allocated to the states one-by-one to the state with the highest claim until all the seats are distributed. It is a general assumption that during the allotment no ties occur, that is all the $\frac{p_{i}}{f(s)}$ values are distinct. In this paper we analyzed the following divisor methods (EP stands for Equal Proportions method—aliases are due to reinventions):

$$\begin{array}{cc} \text{Adams}\;\text{method}\; & f(s)=s\\ \text{Huntington}-\text{Hill}/\text{EP}\;\text{method}\; & f(s)=\sqrt{s(s+1)}\\ \text{Sainte}-\text{Lagu}ë/\text{Webster}\;\text{method}\; & f(s)=s+1/2\\ \text{Jefferson}/\text{D}’\text{Hondt}\;\text{method}\; & f(s)=s+1\\ \text{Imperiali}\;\text{method}\; & f(s)=s+2\\ \text{Macau}\;\text{method}\; & f(s)=2^{s} \end{array}$$

We say that a divisor method is *regular* if the divisor criterion is bounded between *s* and *s* + 1, that is *s* ≤ *f*(*s*) ≤ *s* + 1. Regular divisor methods have a particular feature: Notice that the listed divisor criteria are pointwise increasing—the methods favour large states over small states in the same order. That is, the Adams method favours small states, while the Jefferson/D’Hondt is the most beneficial for large states (see also refs. [20, 22, 23]). Also regular divisor methods may violate either the lower- or the upper-quota, but never both. Non-regular methods like the Imperiali- and Macau methods, may violate the upper- and lower-quota at the same time.

Optimization methods compose the third branch of apportionment methods. The Burt-Harris method [24, 25] minimizes the maximum disparity in representation between any two states, while the *Leximin* method [26], lexicographically minimizes the maximum departure, that is, the difference between the population of any constituency and the average constituency size.

The Venice Commission, the advisory body of the Council of Europe in the field of constitutional law, published The Code of Good Practice in Electoral Matters in 2002 [8], which was consequently used in reviewing Albania’s and Estonia’s electoral law in 2011 [27, 28]. Instead of monotonicity properties this guidebook focuses on the equality of voting power. Optimization methods are the only methods that are conform with the recommendation of the Venice Commission.

There is a slight difference between the recommendation and the Hare-quota requirement. The Hare-quota specifies how many seats a state should receive at least and at most. If a state gets less than its lower quota, then the allotment can be considered somewhat unfair from the point of view of that particular state. The recommendation of the Venice Commission is concerned rather with the individual voter. If the population sizes of the constituencies differ too much so does the voters’ influence. In Europe, where the countries consist of small and in some sense uniform counties the latter makes more sense. Interestingly, the US Supreme Court also ruled that no deviation from equality is too small to challenge as long as a plan with less inequality can be presented (see the case Kirkpatrick v. Preisler (1969)). But this only applies within state. Across states there seems to be no restrictions—this is why currently the voters of Rhode Island have 88% more influence than the voters of Montana [26]. Table 3 summarizes the correspondence between apportionment methods and properties.

**Table 3:** Properties of apportionment methods. ✓ indicates that the solution satisfies, while ✗ indicates that it violates the given property.

Methods	Hare-quota	Monotonicity properties	VC’s recommendation
Largest remainder methods	✓	✗	✗
Divisor methods	✗	✓	✗
Optimization methods	✗	✗	✓

#### Case study of Norway

The choice of apportionment method often depend on cultural and historical characteristics of the country. Even if the decision maker has a clear preference over the three properties (cf. Table 3), each class contains several methods to choose from. Which one performs best on the given data is still up to debate. Since apportionment is also used to distribute seats between parties after the elections, the apportionment method is often challenged in countries with a fragmented parliament. To evaluate the different candidates, malapportionment measures have been proposed [29–33]. SRD follows this literature and offers yet another way to help this difficult choice.

In apportionment, there is a natural candidate for reference point: the respective shares of the states (note that these are non-integer numbers). To demonstrate the effectiveness of SRD we use population data from Norway. In Norway, apportionment is based on the number of voters adjusted by the size of the county. Here we use the raw population data [34] as after adjustment most of the solutions coincide, hence there is no point in comparison. Table 4 shows the sizes of counties, their respective shares and the apportionments proposed by the different methods.

**Table 4:** Comparison of apportionment methods on Norwegian data. Abbreviations: HH: Huntington-Hill, EP: Equal Proportions, SL: Sainte-Laguë.

County	Population	Hamilton/Vinton	Droop	Adams	HH/EP	SL/Webster	Jefferson/D’Hondt	Imperiali	Macau	Burt-Harris	Leximin	Reference
Østfold	282 000	9	9	9	9	9	9	9	9	9	9	9.43
Akershus	566 399	19	20	18	19	19	20	21	10	18	19	18.95
Oslo	623 966	21	21	20	21	21	22	23	10	20	20	20.88
Hedmark	193 719	6	6	7	6	6	6	6	9	7	7	6.48
Oppland	187 254	6	6	6	6	6	6	6	9	6	6	6.26
Buskerud	269 003	9	9	9	9	9	9	9	9	9	9	9.00
Vestfold	238 748	8	8	8	8	8	8	8	9	8	8	7.99
Telemark	170 902	6	6	6	6	6	6	5	9	6	6	5.72
Aust-Agder	112 772	4	4	4	4	4	3	3	8	4	4	3.77
Vest-Agder	176 353	6	6	6	6	6	6	5	9	6	6	5.90
Rogaland	452 159	15	15	15	15	15	16	16	10	15	15	15.13
Hordaland	498 135	17	17	16	17	17	17	18	10	16	16	16.67
Sogn og Fjordane	108 700	4	4	4	4	4	3	3	8	4	4	3.64
Møre og Romsdal	259 404	9	9	9	9	9	9	9	9	9	9	8.68
Sør-Trøndelag	302 755	10	10	10	10	10	10	10	9	10	10	10.13
Nord-Trøndelag	134 443	5	4	5	4	5	4	4	8	5	5	4.50
Nordland	239 611	8	8	8	8	8	8	8	9	8	8	8.02
Troms	160 418	5	5	6	5	5	5	5	8	6	5	5.37
Finnmark	74 534	2	2	3	3	2	2	1	7	3	3	2.49

**Fig 2:**
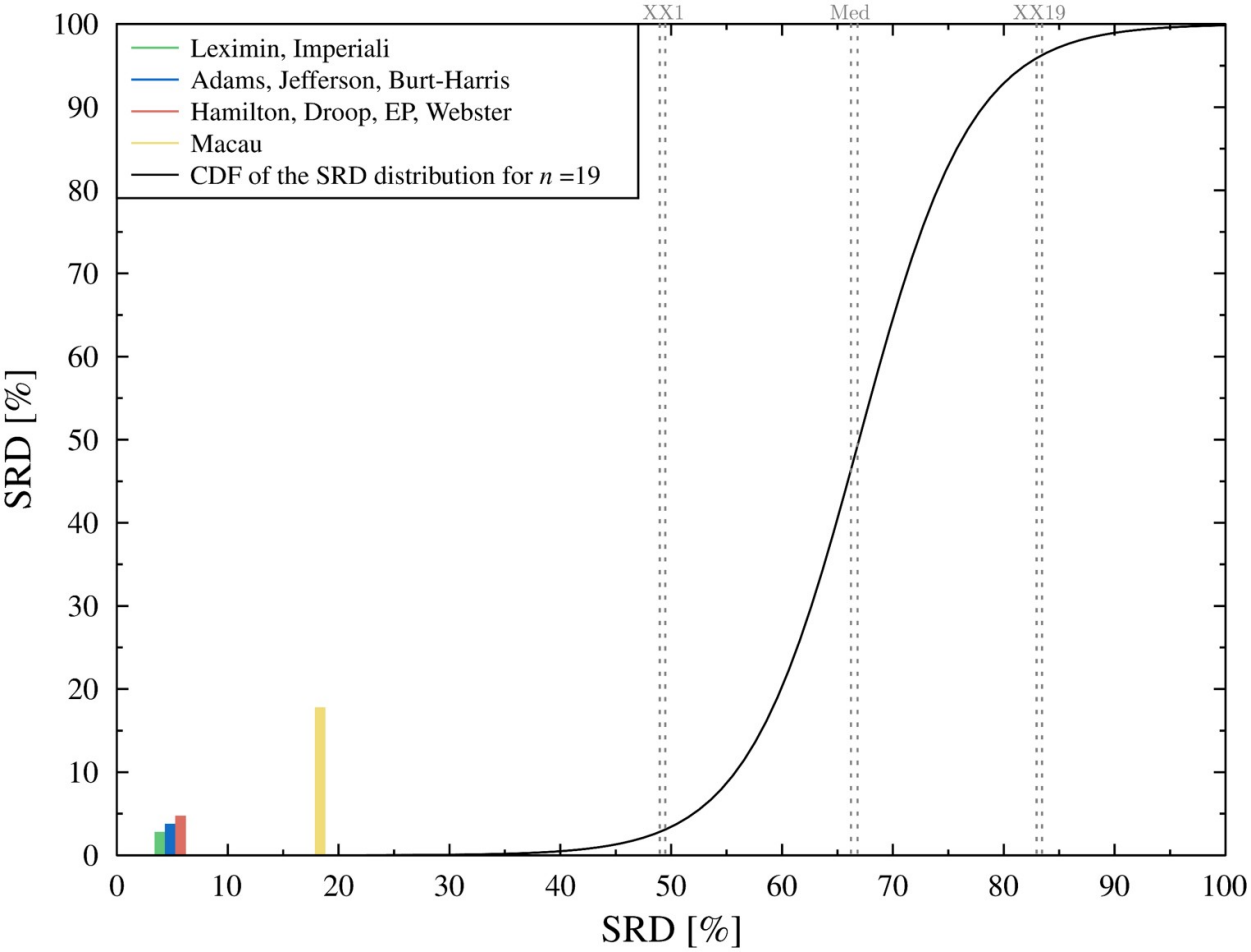
Comparison of ranks with random numbers. All (normalized) SRD values fall outside the 5% threshold (XX1: 5% threshold, Med: Median, XX19: 95% threshold). The black curve is a continuous approximation of the cumulative distribution function of the random SRD values.

The data are typical in the sense, that the solutions prescribed by the different methods are very similar. This is quite common in apportionment, see other examples in refs. [26] or [23]. Given that the apportionment may significantly affect the outcome of the election even small differences matter. The computation of the SRD values are displayed in Table A in S1 Appendix, the results of the CRRN analysis is shown in Fig 2.

With the exception of the exotic Macau method all apportionment methods perform well. Somewhat unexpectedly, the Imperiali method, a non-regular divisor method, shares first place with the Leximin method. This is even more perplexing considering that the Imperiali method does not satisfy *exact quota*. That is, the Imperial method may not produce a perfectly proportional allocation even if such exists. The reason becomes clear when we consider how the Imperiali method handles the quotas. Oslo the largest administrative region gets more seats than its upper quota, while Finnmark, the smallest county gets less than its lower quota. Note, that SRD is insensitive for this kind of bias, the ranking does not change if the largest receives more, or if the smallest obtains less seats. Although the Imperiali seems to favor large states even more than the Jefferson/D’Hondt method, it treats the middle more fairly.

**Fig 3:**
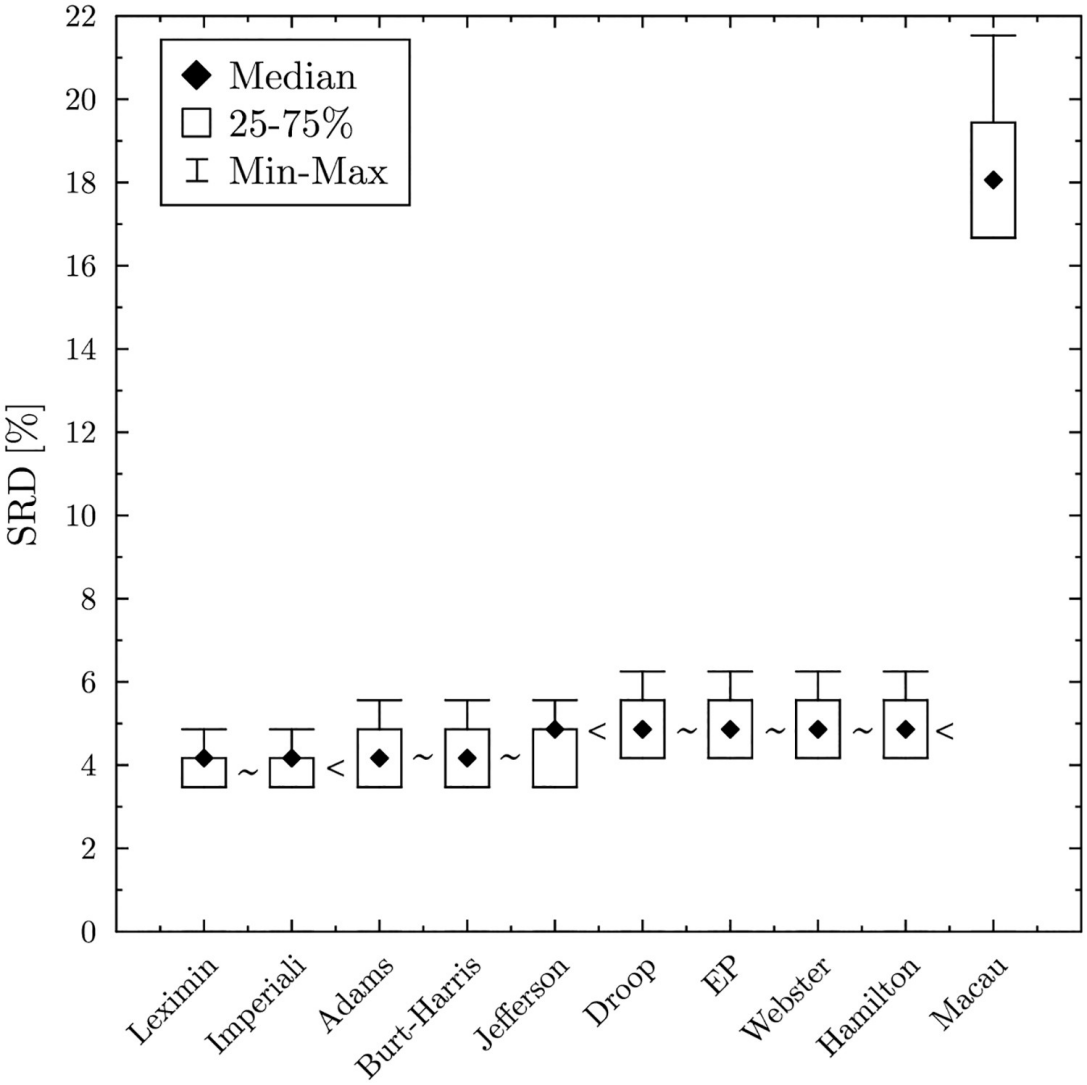
Cross validation—The Wilcoxon test arranges the solutions into four equivalence classes. ‘∼’ indicates that there is no significant difference between the solutions, while ‘<’ indicates that the solutions significantly differ (at the 5% level).

Although the Macau method is inferior compared to the other methods, it still falls outside of the 5% threshold, which means that it is better than a random ranking. Cross-validation also reveals how the solutions are organized (see Fig 3). According to the Wilcoxon test, the Leximin and the Imperiali methods perform significantly better than the Adams or Jefferson/D’Hondt method. The latter two is significantly better, than the EP, Webster and Hamilton methods, while the Macau method lags far behind.

Note that, in the Apportionment problem the rows of the data matrix are dependent in the sense that the total allotment should be equal to the House size. Thus, cross-validation might contain some noise for solutions that are not consistent. Divisor methods are not affected since they are coherent [35], meaning they assign the same allotment for a sub-set of the objects. In Social Choice literature, consistency is the most common expression used to describe this property [36]. In apportionment, the terminology is less consistent and coherence, consistency and uniformity have been equally used. Note that largest remainder methods and optimization methods may not be coherent in this sense. Overall the result should be robust as errors are scarce and cancel out, but for a precise ranking the solutions should be recalculated in each step during the cross-validation.

### Case study No 2: Districting

**Fig 4:**
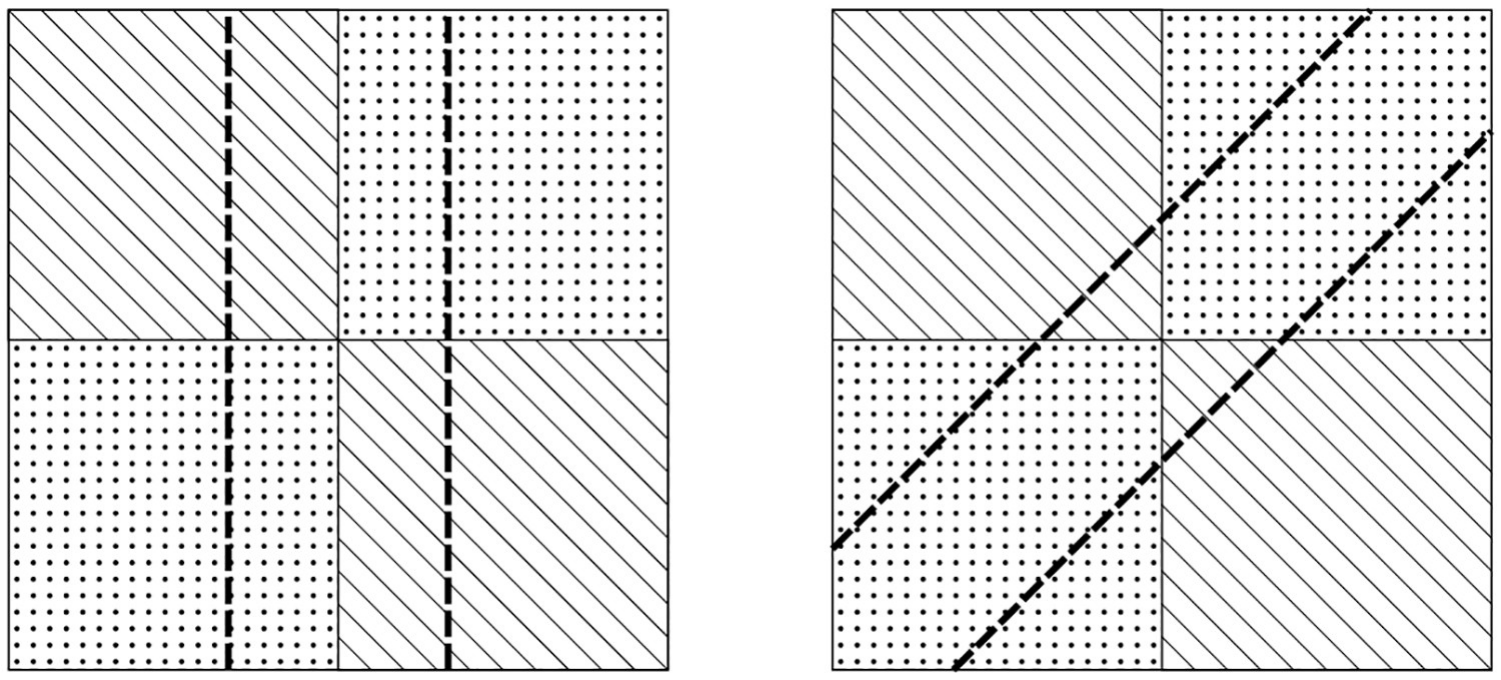
Politically competitive districts (left) *vs*. gerrymandered districts (right).

Ever since US Senator Elbridge Gerry redesigned Essex County’s state senate districts in 1812 to help his re-election, districting is under the spotlight of public attention and there is a continuous academic debate on how legislators ought to do it and how the court should deal with the problematic cases. Fig 4 demonstrates how a politically balanced state can be apportioned to favour one of the parties. Although, here compactness is not an issue, in real life voters are distributed much more erratically and gerrymandered districts tend to have a weird shape.

One of the main questions of districting is whether to construct politically competitive districts, where voters have diverse interests, or to promote proportionality by creating homogeneous districts for all numerically significant sets of political opinions in the electorate [37]. The picture is further complicated by the fact that residential patterns and human geography may cause ‘unintentional gerrymandering’, whereby one party’s voters are more geographically clustered than those of the opposing party [38]. A more recent discussion focuses on how district level competitiveness relates to the marginal benefit of parties’ efforts to mobilize voters, and how competitiveness can be measured [39, 40].

**Fig 5:**
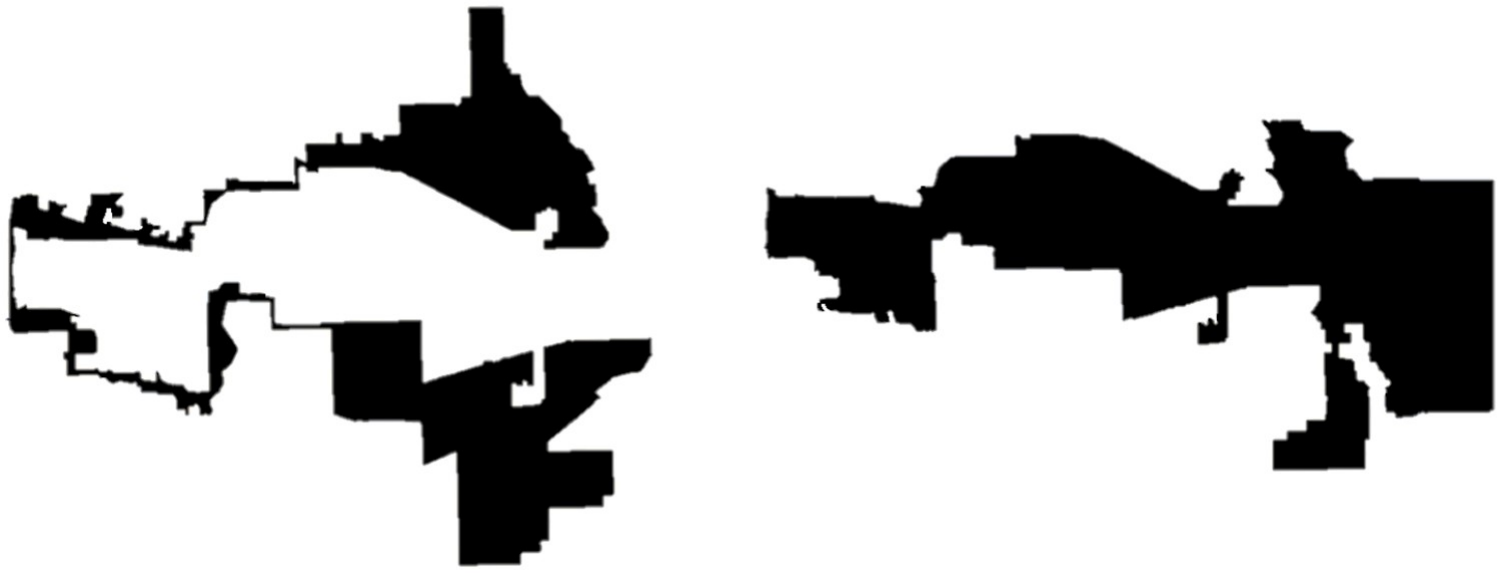
The 4th (left) and 7th (right) congressional districts of Illinois, 108th Congress of the United States.

Perhaps the most controversial case in the US is the ‘earmuffs’ of Chicago, the 4th congressional district of Illinois (Fig 5, left). The constituency which consists of mainly latino voters practically enfolds the 7th district (Fig 5, right), a predominantly black community. The thin line that connects the northern and souther block and ensures the contiguity of the district is an uninhabited highway. The reason (or rather the excuse) of the design is to make sure that both the latino and the black communities are represented in the congress. In reality, this is nothing more than segregation by race, that ignores all cultural aspects: the neighborhood to the north is primarily Puerto Rican, and the one to the south is primarily Mexican-American.

Stern [37] warns, that single-member districts drawn to guarantee minority representation create several problems (*e.g*. the group representing the majority interest in the given district loses incentive for nominating competitive candidates). On the other hand, Gilligan and Matsusaka [41] argue that districting plans that maximize the homogeneity of preferences within each district eliminate policy bias between the median voter and the elected legislature.

It is a delicate issue whether the shapes of the 4th and 7th district of Illinois are justified or not, and discussing it would bring us far from the subject of this paper. Yet, these constituencies give us an excellent idea how hand-drawn districts look like where compactness of the constituency was disregarded.

#### Compactness measures

Compactness has been strongly advocated by legal and political experts as a remedy for partisan gerrymandering [37, 42, 43]. A related stream of literature focuses on measuring redistricting changes, see *e.g*. [44] and the references therein.

There is an intensive debate on what and how compactness measures need to test. For instance, the Iowa Code [45] prescribes that both the length-width difference and the perimeter of a district should be minimal and the total length-width difference and the total perimeter distance computed for all individual districts in a plan can be compared to an alternative districting plan.

In contrast, many compactness measures compare the shape of a constituency to an ideal formation: a circle or rectangle. There are other approaches: Chambers and Miller [46] suggest a path-based measure without specifying an ideal form. Here we review some of the classical measures as well as a novel method recommended recently by Nagy and Szakál [47].

The Polsby-Popper test [42] compares the area of the district to the area of a circle with the same perimeter as the district. The Reock test [48] compares the area of the district to the area of the smallest circle within which the district will fit. The Lee-Sallee test [49] again considers a circle with the same area as the district and places it in such way that the center of mass of the two shapes coincides, then takes the ratio of the area of their intersection and the area of their union. The Moment Invariants comes from image processing and also considers the circle the most compact shape. The Length-to-width test takes the (absolute) difference between the distances of the Westernmost and Easternmost points and the Southernmost and Northernmost points of the district [50].

Formally, let D represent the set of geometric shapes corresponding to the constituencies. We denote the area of a constituency D∈D as *A*(*D*), while let *P*(*D*) be its perimeter. Furthermore, let *C*′ be the smallest circumscribed circle of *D*, and *C*″ a circle such that *A*(*C*′) = *A*(*D*), and the center of mass for *C*″ and *D* coincides.

$$\begin{array}{cc} \text{Polsby-Popper}\; & C_{PP}\left(D\right)=\frac{4\pi \cdot A(D)}{{\left(P\left(D\right)\right)}^{2}}\\ \text{Reock} & \;C_{R}\left(D\right)=\frac{A(D)}{A(C^{\prime })}\\ \text{Lee-Sallee}\; & C_{LS}\left(D\right)=\frac{A(D\cap C^{\prime \prime })}{A(D\cup C^{\prime \prime })}\\ \text{Moment}\;\text{Invariants}\; & C_{MI}^{\beta }\left(D\right)=\{\begin{array}{cc} \frac{{\left(A\left(D\right)\right)}^{\beta +1}}{\pi^{\beta }\left(\beta +1\right)\int \int_{D}{\left(x^{2}+y^{2}\right)}^{\beta }dxdy}, & \text{if}\;\beta >0\\ \frac{\pi^{\beta }\left(\beta +1\right)\int \int_{D}{\left(x^{2}+y^{2}\right)}^{\beta }dxdy}{{\left(A\left(D\right)\right)}^{\beta +1}}, & \text{if}\;\beta \in (-1,0). \end{array} \end{array}$$

All the above measures range between 0 and 1, and $C_{PP}\left(D\right)=C_{R}\left(D\right)=C_{LS}\left(D\right)=C_{MI}^{\beta }\left(D\right)=1\Leftrightarrow D$ is a circle. To make the Length-to-width measure comparable to the other measures we transform the values into the [0, 1] interval. Let *LW*(*D*) stand for the length-width difference of district *D*, then we standardize the data with the following formula

$$\begin{array}{cc} \text{Length-to-width}\; & C_{LW}\left(D\right)=1-\frac{LW(D)}{max_{D^{\prime }\in D}\left\{LW\left(D^{\prime }\right)\right\}}. \end{array}$$

#### Comparing compactness measures

**Fig 6:**
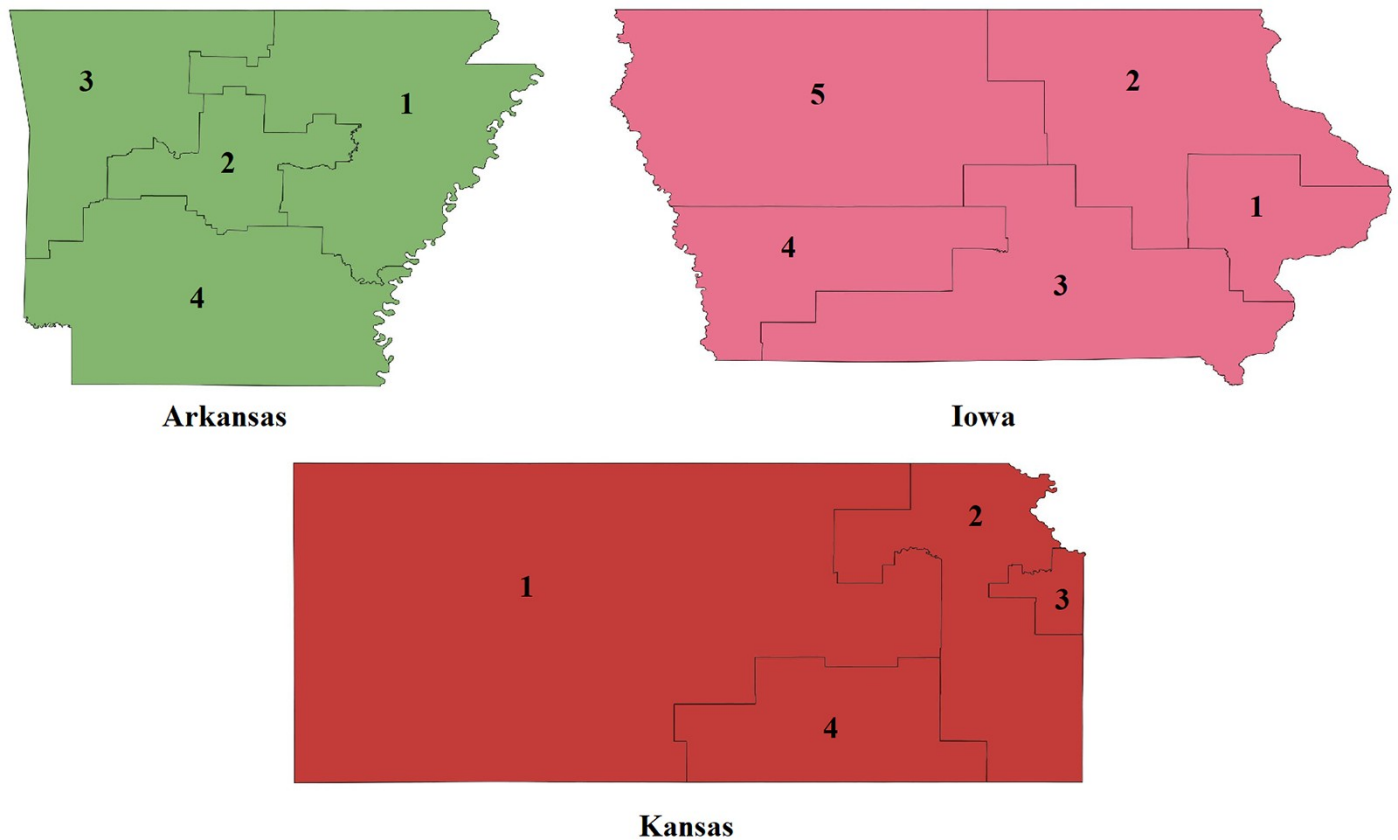
Congressional districts of Arkansas, Iowa and Kansas, 107th Congress (Source: [51]).

To test how compactness measures perform on real data, we use the dataset provided in [47], where compactness of the congressional districts of Arkansas, Iowa and Kansas are compared (Table 5 and Fig 6). Unlike to the apportionment problem, in districting there is no natural reference point. Since the ideal shape, a circle, has a compactness measure of 1, and generally the greater the value the more compact the shape is, the best (maximum) or worst (minimum) values could be potential reference points. Notice however, that although all compactness measures map into [0, 1], some of them have a preferred subinterval. For instance, the Lee-Sallee index ranges between 0.4 and 0.8, while the Polsby-Popper between 0.1 and 0.5. Choosing the maximum values would effectively result in setting the Moment Invariants with (*β* = −0.5) as the reference. The minimum values are no better as they almost always coincide with the Polsby-Popper scores. Hence, in this case, the minimum or maximum values do not allow the objective comparison of these measures. Instead we opt for a third candidate and set the average as the reference point.

**Table 5:** Compactness measures for various congressional districts of the 107th Congress of the United States (Source: [47] and own compilation).

Districts	Mom. Inv. (*β* = −0.5)	Mom. Inv. (*β* = 1)	Mom. Inv. (*β* = 2)	Lee-Sallee	Reock	Polsby-width	Length-to-(Avg)	Reference
Arkansas 1st	0.936	0.810	0.584	0.721	0.396	0.144	0.924	0.645
Arkansas 2nd	0.924	0.640	0.301	0.582	0.311	0.221	0.693	0.524
Arkansas 3rd	0.940	0.698	0.365	0.619	0.328	0.327	0.824	0.586
Arkansas 4th	0.947	0.753	0.474	0.617	0.394	0.260	0.292	0.534
Iowa 1st	0.944	0.790	0.527	0.655	0.388	0.403	0.980	0.670
Iowa 2nd	0.895	0.504	0.170	0.483	0.208	0.255	0.720	0.462
Iowa 3rd	0.881	0.544	0.224	0.445	0.254	0.302	0.025	0.382
Iowa 4th	0.948	0.758	0.483	0.610	0.428	0.468	0.549	0.606
Iowa 5th	0.945	0.729	0.399	0.654	0.273	0.323	0.418	0.534
Kansas 1st	0.950	0.734	0.430	0.790	0.387	0.431	0.000	0.532
Kansas 2nd	0.854	0.577	0.298	0.439	0.355	0.230	0.353	0.443
Kansas 3rd	0.910	0.743	0.472	0.619	0.389	0.355	0.942	0.633
Kansas 4th	0.923	0.655	0.332	0.549	0.346	0.467	0.343	0.516

Theoretical and practical arguments equally support this choice. Firstly, if we think of the compactness measures as tests that estimate compactness with some error, then by taking the average these errors cancel out by the maximum likelihood principle. The fact that the measures capture completely different aspects of compactness actually strengthen this point, as it is less likely that the average is affected by some kind of systematic bias. Secondly, if a policy maker has to decide which measure to impose as a legal requirement, she might prefer to choose something close to the average as she doesn’t want her decision to be challenged.

**Fig 7:**
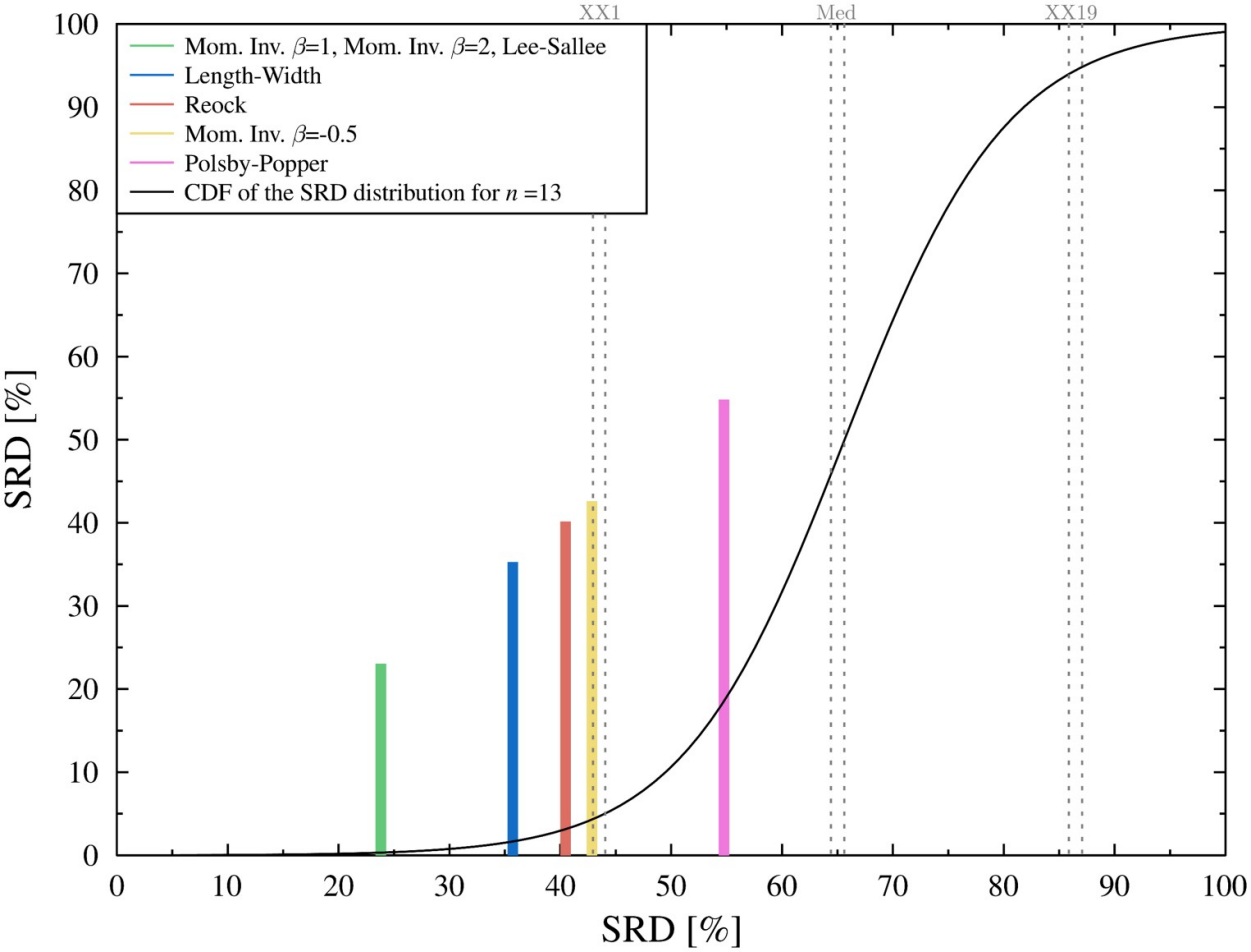
Comparison of ranks with random numbers (cf. Fig 2). Great distance between the SRD values.

Table B of S1 Appendix displays the computation of SRD values, Fig 7 shows the result of CRRN test. There are a couple of interesting observations to make. In contrast to apportionment, here we see great distances between the SRD values. Moreover, even the best SRD scores are not that good—there is room for improvement. The Moment Invariant measures obtained some of the best and worst SRD scores, which indicates that the parameter of the function should be chosen carefully. Nagy and Szakál [47] suspect, based on empirical observations, that the most effective interval for the *β* parameter is [1, 3]. Indeed *β* = −0.5 is second worst among the solutions and just barely falls outside the error limit. Finally, the most apparent feature is that the Polsby-Popper test falls within the error limit, that is, it cannot be distinguished from random ranking.

**Fig 8:**
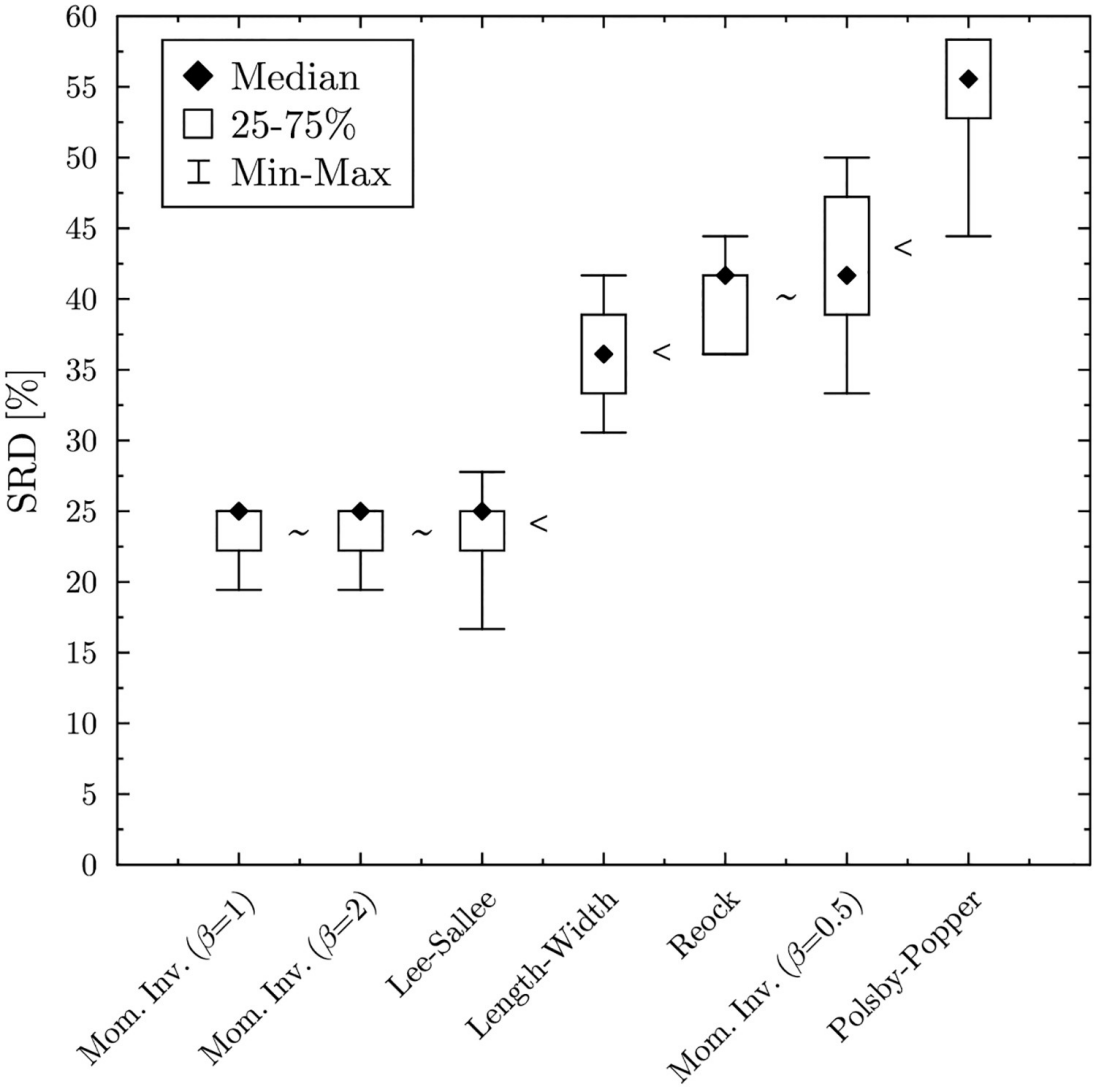
Cross validation. ‘∼’ indicates that there is no significant difference between the solutions, while ‘<’ indicates that the solutions significantly differ.

Cross-validation (Fig 8) confirms that there is no significant difference between Moment Invariants with *β* = 1 and *β* = 2 and the Lee-Sallee index. The triumvirate is followed by the Length-to-width test, then by the Reock test and Moment Invariant with *β* = −0.5, which again do not differ significantly. Finally comes, lagging somewhat behind, the Polsby-Popper test. In this case, objects are independent, no consistency-issues arise.

Arguably, measuring compactness is a complicated, multi-dimensional problem. The bad SRD score of the Polsby-Popper test might only indicate that this test measures a different aspect of the problem. On the other hand, this can be said basically about every other tests. Table 6 summarizes the relative distances of the solutions. One-by-one, we fixed each solution as the reference and computed the distances in SRD scores for each other solution. The Polsby-Popper test, as expected, is quite far away from the other measures, but so is the Length-to-width test, and none of them seems to be particularly close to each other (with the exception of the Moment Invariants with *β* = 1 and *β* = 2). If we think of the average value as a collective wisdom that reflects the judgment of all the measures, then one may be inclined to say that the Polsby-Popper test is unsuitable for measuring compactness of constituencies. We do not wish to formulate such a strong claim. A sample of 13 districts is hardly big enough to make such a generalization. Further analysis is needed to resolve this issue.

**Table 6:** The relative heat map shows the distances between solutions measured in SRD score, when the reference is set one-by-one as one of the solutions.

	Mom. Inv. β = 1	Mom. Inv. β = 2	Lee-Sallee	Reock	Mom. Inv. β = -0.5	Polsby-Popper	Length-to-width
Mom. Inv. β = 1	0.00	0.00	30.95	21.43	38.10	50.00	52.38
Mom. Inv. β = 2	0.00	0.00	30.95	21.43	38.10	50.00	52.38
Lee-Sallee	30.95	30.95	0.00	47.62	28.57	52.38	50.00
Reock	21.43	21.43	47.62	0.00	45.24	57.14	61.90
Mom. Inv. β = -0.5	38.10	38.10	28.57	45.24	0.00	50.00	71.43
Polsby-Popper	50.00	50.00	52.38	57.14	50.00	0.00	73.81
Length-to-width	52.38	52.38	50.00	61.90	71.43	73.81	0.00
**Colour code**:	**x<=**	7.4	14.8	22.1	29.5	36.9	
		44.3	51.7	59.0	66.4	73.8	

Nevertheless, this result has a practical consequence. Policy makers that seek to reform districting law and impose a compactness requirement might be less inclined to propose the Polsby-Popper test. Since the test’s measurements are off from the average, its results can be easily challenged by an adverse party armed with a different measure.

## Summary and conclusion

Sum of Ranking Differences is a novel statistical method, which can be valuable for testing competing solutions in Political Science and Social Choice. We provided two case studies to demonstrate its effectiveness in fair division and fair assessment problems. For the former we looked at the apportionment of the Norwegian parliamentary seats. For the latter we considered the compactness of the constituencies of three US states.

In the apportionment problem, all the methods under examination—with the exception of the Macau-method—performed very well. Although the Leximin method fit the data the best, it was only slightly better than classical solution methods. Overall, optimization methods produced better SRD values than largest remainder methods. However, to announce a clear ranking of the methods more tests are needed. Interestingly, the non-regular Imperiali method performed just as well as the Leximin method. The likely cause is, that the Imperiali method may violate both the upper- and lower quota in the same time, and SRD does not penalize this behavior. Still, the outstanding SRD score indicates that the Imperiali method is not just another exotic apportionment method, but a viable alternative to the classical rules.

In the districting problem, the SRD values covered a far greater range. A novel parametric method, the Moment Invariants performed very well compared to the classical compactness measures when the parameter was chosen carefully, that is for *β* = 1 and *β* = 2. However, for *β* = −0.5 the method fares poorly. The SRD score of the Polsby-Popper test was no better than an SRD value of a random ranking, which suggests that the test measures a different dimension of compactness. Further analysis is needed to decide whether the test is suitable for measuring the compactness of constituencies. In reality, the reliability of compactness measures are limited as they do not take into account the natural boundaries (*e.g*. coastlines). This can be avoided by looking at the redistricting problem on a higher level and compare total compactness of competing redistricting plans.

In summary, SRD seems to be an excellent tool in comparing solutions in various fields of applied science. Initial steps has been already taken to provide theoretical foundations for its success. Lourenço and Lebensztajn [4] showed that SRD provides a smaller set of optimal solutions from among the possible groupings of similar solutions of the Pareto front. An axiomatic analysis of SRD would further strengthen its reliability.

## Supporting information

S1 Appendix Computation of SRD values in the apportionment problem and Computation of SRD values in the districting problem. (PDF)
