# Supplementary material for: Apportionment and districting by Sum of Ranking Differences
Source: PLoS One. 2020 Mar 23;15(3):e0229209. doi: 10.1371/journal.pone.0229209 (PMC7089544; doi:10.1371/journal.pone.0229209)
Supplement: S1 Appendix — (PDF) [file pone.0229209.s001.pdf]

Apportionment and districting by Sum of Ranking Differences

Balázs R. Sziklai[1,2*], Károly Héberger[3]

1 Institute of Economics, Centre for Economic and Regional Studies, Hungary
2 Department of Operations Research and Actuarial Sciences, Corvinus University of Budapest, Hungary
3 Institute of Materials and Environmental Chemistry, Research Centre for Natural Sciences, Hungary

* sziklai.balazs@krtk.mta.hu

S1 Appendix

Computation of SRD values in the apportionment problem

County	Hamilton	Diff.	Droop	Diff.	Adams	Diff.	EP	Diff.	Webster	Diff.	Reference ranking
Finnmark	1	0	1	0	1	0	1	0	1	0	1
Sogn og Fjordane	2.5	0.5	3	1	2.5	0.5	3	1	2.5	0.5	2
Aust-Agder	2.5	0.5	3	0	2.5	0.5	3	0	2.5	0.5	3
Nord-Trøndelag	4.5	0.5	3	1	4	0	3	1	4.5	0.5	4
Troms	4.5	0.5	5	0	6.5	1.5	5	0	4.5	0.5	5
Telemark	7.5	1.5	7.5	1.5	6.5	0.5	7.5	1.5	7.5	1.5	6
Vest-Agder	7.5	0.5	7.5	0.5	6.5	0.5	7.5	0.5	7.5	0.5	7
Oppland	7.5	0.5	7.5	0.5	6.5	1.5	7.5	0.5	7.5	0.5	8
Hedmark	7.5	1.5	7.5	1.5	9	0	7.5	1.5	7.5	1.5	9
Vestfold	10.5	0.5	10.5	0.5	10.5	0.5	10.5	0.5	10.5	0.5	10
Nordland	10.5	0.5	10.5	0.5	10.5	0.5	10.5	0.5	10.5	0.5	11
Møre og Romsdal	13	1	13	1	13	1	13	1	13	1	12
Buskerud	13	0	13	0	13	0	13	0	13	0	13
Østfold	13	1	13	1	13	1	13	1	13	1	14
Sør-Trøndelag	15	0	15	0	15	0	15	0	15	0	15
Rogaland	16	0	16	0	16	0	16	0	16	0	16
Hordaland	17	0	17	0	17	0	17	0	17	0	17
Akershus	18	0	18	0	18	0	18	0	18	0	18
Oslo	19	0	19	0	19	0	19	0	19	0	19
SRD values		9		9		8		9		9	

County	Jefferson	Diff.	Imperiali	Diff.	Macau	Diff.	Burt-Harris	Diff.	Leximin	Diff.	Reference ranking
Finnmark	1	0	1	0	1	0	1	0	1	0	1
Sogn og Fjordane	2.5	0.5	2.5	0.5	3.5	1.5	2.5	0.5	2.5	0.5	2
Aust-Agder	2.5	0.5	2.5	0.5	3.5	0.5	2.5	0.5	2.5	0.5	3
Nord-Trøndelag	4	0	4	0	3.5	0.5	4	0	4.5	0.5	4
Troms	5	0	6	1	3.5	1.5	6.5	1.5	4.5	0.5	5
Telemark	7.5	1.5	6	0	10.5	4.5	6.5	0.5	7	1	6
Vest-Agder	7.5	0.5	6	1	10.5	3.5	6.5	0.5	7	0	7
Oppland	7.5	0.5	8.5	0.5	10.5	2.5	6.5	1.5	7	1	8
Hedmark	7.5	1.5	8.5	0.5	10.5	1.5	9	0	9	0	9
Vestfold	10.5	0.5	10.5	0.5	10.5	0.5	10.5	0.5	10.5	0.5	10
Nordland	10.5	0.5	10.5	0.5	10.5	0.5	10.5	0.5	10.5	0.5	11
Møre og Romsdal	13	1	13	1	10.5	1.5	13	1	13	1	12
Buskerud	13	0	13	0	10.5	2.5	13	0	13	0	13
Østfold	13	1	13	1	10.5	3.5	13	1	13	1	14
Sør-Trøndelag	15	0	15	0	10.5	4.5	15	0	15	0	15
Rogaland	16	0	16	0	17.5	1.5	16	0	16	0	16
Hordaland	17	0	17	0	17.5	0.5	17	0	17	0	17
Akershus	18	0	18	0	17.5	0.5	18	0	18	0	18
Oslo	19	0	19	0	17.5	1.5	19	0	19	0	19
SRD values		8		7		33		8		7	

Table A. Rankings induced by the various solutions and difference from the reference ranking

Computation of SRD values in the districting problem

Districts	Mom. Inv. ($\beta = -0.5$)	Diff.	Mom. Inv. ($\beta = 1$)	Diff.	Mom. Inv. ($\beta = 2$)	Diff.	Lee-Sallee	Diff.	Reference ranking
Iowa 3rd	2	1	2	1	2	1	2	1	1
Kansas 2nd	1	1	3	1	3	1	1	1	2
Iowa 2nd	3	0	1	2	1	2	3	0	3
Kansas 4th	5	1	5	1	5	1	4	0	4
Arkansas 2nd	6	1	4	1	4	1	5	0	5
Kansas 1st	13	7	8	2	8	2	13	7	6
Arkansas 4th	11	4	10	3	10	3	7	0	7
Iowa 5th	10	2	7	1	7	1	10	2	8
Arkansas 3rd	8	1	6	3	6	3	8	1	9
Iowa 4th	12	2	11	1	11	1	6	4	10
Kansas 3rd	4	7	9	2	9	2	9	2	11
Arkansas 1st	7	5	13	1	13	1	12	0	12
Iowa 1st	9	4	12	1	12	1	11	2	13
SRD value		18		10		10		10	

	Reock	Diff.	Polsby-Popper	Diff.	Length-to-width	Diff.	Reference ranking
Iowa 3rd	2	1	6	5	2	1	1
Kansas 2nd	7	5	3	1	5	3	2
Iowa 2nd	1	2	4	1	9	6	3
Kansas 4th	6	2	12	8	4	0	4
Arkansas 2nd	4	1	2	3	8	3	5
Kansas 1st	8	2	11	5	1	5	6
Arkansas 4th	11	4	5	2	3	4	7
Iowa 5th	3	5	7	1	6	2	8
Arkansas 3rd	5	4	8	1	10	1	9
Iowa 4th	13	3	13	3	7	3	10
Kansas 3rd	10	1	9	2	12	1	11
Arkansas 1st	12	0	1	11	11	1	12
Iowa 1st	9	4	10	3	13	0	13
SRD value		17		23		15	

Table B. Rankings induced by the various solutions and difference from the reference ranking
